# A Genetic Deconstruction of Neurocognitive Traits in Schizophrenia and Bipolar Disorder

**DOI:** 10.1371/journal.pone.0081052

**Published:** 2013-12-12

**Authors:** Carla P. D. Fernandes, Andrea Christoforou, Sudheer Giddaluru, Kari M. Ersland, Srdjan Djurovic, Manuel Mattheisen, Astri J. Lundervold, Ivar Reinvang, Markus M. Nöthen, Marcella Rietschel, Roel A. Ophoff, Genetic Risk and Outcome of Psychosis (GROUP), Albert Hofman, André G. Uitterlinden, Thomas Werge, Sven Cichon, Thomas Espeseth, Ole A. Andreassen, Vidar M. Steen, Stephanie Le Hellard

**Affiliations:** 1 K. G. Jebsen Centre for Psychosis Research and the Norwegian Centre for Mental Disorders Research (NORMENT), Department of Clinical Science, University of Bergen, Bergen, Norway; 2 Dr. Einar Martens Research Group for Biological Psychiatry, Center for Medical Genetics and Molecular Medicine, Haukeland University Hospital, Bergen, Norway; 3 K. G. Jebsen Centre for Psychosis Research, Norwegian Centre For Mental Disorders Research (NORMENT), Division of Mental Health and Addiction, Oslo University Hospital & Institute of Clinical Medicine, University of Oslo, Oslo, Norway; 4 Department of Genomics, Life & Brain Center, University of Bonn, Bonn, Germany; 5 Channing Division of Network Medicine, Brigham and Women's Hospital and Harvard Medical School, Boston, Massachusetts, United States of America; 6 Institute for Genomic Mathematics, University of Bonn, Bonn, Germany; 7 Department of Biological and Medical Psychology, University of Bergen, Bergen, Norway; 8 Kavli Research Centre for Aging and Dementia, Haraldsplass Deaconess Hospital, Bergen, Norway; 9 K. G. Jebsen Centre for Research on Neuropsychiatric Disorders, University of Bergen, Bergen, Norway; 10 Department of Psychology, University of Oslo, Oslo, Norway; 11 Institute of Human Genetics, University of Bonn, Bonn, Germany; 12 German Center for Neurodegenerative Diseases (DZNE), Bonn, Germany; 13 Department of Genetic Epidemiology in Psychiatry, Central Institute of Mental Health, Medical Faculty Mannheim, University of Heidelberg, Heidelberg, Germany; 14 Department of Psychiatry, Rudolf Magnus Institute of Neuroscience, University Medical Center Utrecht, Utrecht, The Netherlands; 15 Center for Neurobehavioral Genetics, University of California Los Angeles, Los Angeles, California, United States of America; 16 Department of Epidemiology, Erasmus University Medical Center, Rotterdam, The Netherlands; 17 Department of Internal Medicine, Genetics Laboratory, Erasmus Medical Center Rotterdam, Rotterdam, The Netherlands; 18 Mental Health Centre Sct. Hans, Copenhagen University Hospital, Research Institute of Biological Psychiatry, Roskilde, Denmark; 19 Institute of Neuroscience and Medicine (INM-1), Research Center Juelich, Juelich, Germany; 20 Division of Mental Health and Addiction, Oslo University Hospital and Institute of Clinical Medicine, University of Oslo, Oslo, Norway; University of Illinois at Chicago, United States of America

## Abstract

**Background:**

Impairments in cognitive functions are common in patients suffering from psychiatric disorders, such as schizophrenia and bipolar disorder. Cognitive traits have been proposed as useful for understanding the biological and genetic mechanisms implicated in cognitive function in healthy individuals and in the dysfunction observed in psychiatric disorders.

**Methods:**

Sets of genes associated with a range of cognitive functions often impaired in schizophrenia and bipolar disorder were generated from a genome-wide association study (GWAS) on a sample comprising 670 healthy Norwegian adults who were phenotyped for a broad battery of cognitive tests. These gene sets were then tested for enrichment of association in GWASs of schizophrenia and bipolar disorder. The GWAS data was derived from three independent single-centre schizophrenia samples, three independent single-centre bipolar disorder samples, and the multi-centre schizophrenia and bipolar disorder samples from the Psychiatric Genomics Consortium.

**Results:**

The strongest enrichments were observed for visuospatial attention and verbal abilities sets in bipolar disorder. Delayed verbal memory was also enriched in one sample of bipolar disorder. For schizophrenia, the strongest evidence of enrichment was observed for the sets of genes associated with performance in a colour-word interference test and for sets associated with memory learning slope.

**Conclusions:**

Our results are consistent with the increasing evidence that cognitive functions share genetic factors with schizophrenia and bipolar disorder. Our data provides evidence that genetic studies using polygenic and pleiotropic models can be used to link specific cognitive functions with psychiatric disorders.

## Introduction

Schizophrenia (SCZ) and bipolar disorder (BPD) are devastating major psychiatric disorders that affect approximately 1% of the population worldwide in a lifetime perspective [1]. They are characterized by prominent clinical symptoms such as delusions and hallucinations for SCZ, or mania and depression in BPD. In both disorders, impairments in cognitive function are often observed, which play major roles in the intellectual and social dysfunction of the patients [2], [3]. Although these impairments are usually stronger in patients, the deficits are also observed in their unaffected relatives; for instance, deficits across several cognitive domains such as learning, memory and executive function have been observed in unaffected relatives of patients with SCZ [4] or in their unaffected twin siblings [5]. These studies have shown that a substantial amount of variance in cognitive abilities and impairments is due to shared genetic effects between the cognitive abilities and the psychiatric disorders [6]. It has been suggested that measures of these neurocognitive functions represent underlying phenotypes (or intermediate phenotypes) in patients [7]. They also represent quantitative phenotypes in healthy individuals that may help characterize the behavioral traits, biological functions and genetic factors underlying major psychiatric disorders [8], [9].

Genetic susceptibility plays a major role in SCZ and BPD but the identification of genetic factors is obscured by a complex and polygenic architecture [10], [11]. While the population estimates of heritability are 64% for SCZ and 59% for BPD [12], the genetic basis or “heritability” that can be explained by common variants genotyped in current GWASs [13] is up to 30% and 40%, respectively. However classical *p*-value threshold-based GWAS analyses capture only a small number of variants that together explain less than 3% of this genetic basis [11] even in large samples. Thus, alternative approaches to GWAS analysis are needed to capture the so called “hidden heritability”, especially the 30–40% explained by common factors that are not captured by threshold-based analyses.

Typically in classical GWAS analysis, because of the high number of genetic variants tested, a conservative *p*-value threshold of 5×10^−8^ is applied, at the cost of losing many variants of small effect that are truly implicated in the trait being studied. In contrast, polygenic methods include genetic variants with smaller effect (i.e. genetic variants that do not pass the threshold) and evaluate these variants as a group for their effect on a trait. These methods can be categorized into two main groups, namely marker-based methods and gene-based methods. Gene-based methods offer the additional advantage of being more permissive to allelic heterogeneity, whereby several independent variants at the same functional locus can have an effect on the same trait, or across traits if several phenotypes are being compared. Allelic heterogeneity is well documented in complex traits. For instance, in a recent re-analysis of GWAS data, Yang *et al*. [14] showed that several variants within a locus were associated independently with height and body mass index. *DISC1* [15], *DCLK1* [16], [17], *TCF4* [18], [19], *NPAS3* [20], [21], and *CSMD2* [22], [23] are examples of genes for which associations with psychiatric disorders and cognitive traits have been described but with different markers in the gene. Even though type I and II errors could explain these observations, most of the differences observed between samples and traits are probably explained by allelic heterogeneity occurring because several functional variants in a gene have an effect, or because the genetic structure varies between samples, or because there is imperfect tagging of the causal variation by the markers typed [24]. Thus methods that account for allelic heterogeneity, such as gene-based approaches, are better adapted to compare association across samples and across traits than single-marker methods.

Since impaired cognitive abilities are core features of SCZ and BPD, genetic factors implicated in cognitive abilities are likely to overlap with the genetic variants implicated in disease risk [9], [25]. There is now an increased interest in investigating the genetic overlap between cognitive functions and psychiatric disorders with polygenic methods. For instance, in a recently published study, McIntosh *et al*.[26] show that a polygenic risk of SCZ calculated from whole-genome variation was associated with lower IQ at age 70 and greater decline in IQ level. In the present study, we used a gene-based approach to try to identify which cognitive functions, from a selection of domains that have been reported as impaired in SCZ and BPD, show the strongest overlap with these disorders at the gene level. We chose a gene-based method in order to integrate the effect of allelic heterogeneity, which is not accounted for in marker-based polygenic studies. We generated sets of genes associated with nine different cognitive tests in healthy individuals, then tested these sets for enrichment in GWASs of SCZ and BPD. Our most significant finding was that sets of genes associated with visuospatial attention and verbal abilities were the most significantly enriched in the BPD samples and the sets of genes associated with performance in a colour-word interference test and with the learning slope in a memory task were enriched in SCZ samples.

## Materials and Methods

### Description of the Samples

#### Ethics Statement

The work described here was approved by the regional ethical committee for medical research (Project ID: S-03116) for the NCNG sample and the relevant national ethical committees for the different samples of patients with psychiatric disorders. Written consent was obtained from all participating individuals before initiating the study.

#### The Norwegian Cognitive NeuroGenetics Sample

The Norwegian Cognitive NeuroGenetics sample (NCNG) consists of 670 healthy Norwegian subjects extensively tested for different cognitive abilities, which are described in detail in the protocol paper from Espeseth *et al.* [27] (see also Table S1 in File S1). The participants comprise 457 females and 213 males, with a mean age of 47.6 (range 18–79 years) recruited in Oslo (n = 499) and Bergen (n = 171). We selected nine cognitive tests that we consider to best represent each one of the different cognitive domains relevant to psychiatric disorders as reported in the literature [25], [28]–[34]. We generated an estimated Intelligence Quotient (cognitive function “**Estimated IQ**”) from the Vocabulary (cognitive domain “**Verbal abilities**”) and Matrix Reasoning (cognitive function “**Matrix reasoning**”) sub-tests from the Wechsler Abbreviated Scale of Intelligence [35]. From the California Verbal Learning Test – second edition [36] we used the total number of words learned across five trials as a measure for learning (cognitive function “**Learning**”); the delayed free recall score (cognitive function “**Delayed verbal memory**”); the third condition from the D-KEFS Colour-Word Interference Test (Stroop3) (cognitive function “**Colour-word interference**”) [37]; and the valid, invalid and neutral conditions of the Cued Discrimination Task (cognitive functions “**Visuospatial attention 1/2/3**” respectively) [38]. Although the three visuospatial attention traits are highly correlated at the phenotypic level, they still correspond to different cognitive processes (for example, the ability to redirect attention when the cue is invalid); thus we chose to test the three processes independently.

The NCNG sample was genotyped using the Illumina Human 610-Quad BeadChip, retaining 554,225 SNPs after stringent quality control, performed using the GenABEL package [39]. Duplicated samples or those from closely related individuals – identity-by-state threshold ≥0.85 – were excluded. Individual samples were removed if the heterozygosity values were greater than two standard deviations (*SD*s) (z-test two-tailed *P* = 0.05) from the sample mean or if they had sex discrepancies. Since we aimed at a genetically homogenous sample at the population level to decrease the genetic heterogeneity, the population structure was assessed by multidimensional scaling (MDS) analysis using 100K random single nucleotide polymorphisms (SNPs) to exclude possible non-Norwegian ancestors (24 samples were excluded). SNPs were filtered and excluded from the analysis if they had a call rate <0.95, minor allele frequency (MAF) <0.01 and Hardy-Weinberg Equilibrium (HWE) exact test P <0.001. For further details about the NCNG sample and genotyping quality control, see Espeseth *et al.* [27] and Davies *et al*. [40].

The neurocognitive traits were analyzed using linear regression, as implemented in PLINK (http://pngu.mgh.harvard.edu/purcell/plink/) [41], using sex and age as covariates (except for estimated IQ which was already corrected for age).

#### Psychiatric Disorder Samples

Three independent GWASs for SCZ were tested (Table 1): a combined German-Dutch GWAS [42], the Danish sub-sample of the Scandinavian Collaboration on Psychiatric Etiology (SCOPE) [43], and the Norwegian Thematically Organized Psychosis (TOP) Study SCZ sample (extended since the original publication [44]). We also tested the SCZ multi-centre sample from the Psychiatric Genomics Consortium (PGC) [19]. 20% of the cases and 22% of the controls from the PGC sample overlap with the other three samples (Table 1).

**Table 1:** Description of the samples.

Phenotype	Sample	Cases	Controls	Cases/controls in PGC*	Genotyping platform
**Healthy**	NCNG		670		Illumina Human610-Quad
**BPD**	Norwegian-TOP	575	417	203/349	Affymetrix Genome-Wide Human SNP Array 6.0
	German	682	1300	675/1297	Illumina HumanHap550v3
	WTCCC	1868	2938	1571/2931	Affymetrix GC500K
	PGC	7481	9250		Several (see ref. 19)
**SCZ**	Norwegian-TOP	405	417	248/351	Affymetrix Genome-Wide Human SNP Array 6.0
	German-Dutch	1169	3714	1178/1935	Illumina HumanHap550v3
	Danish	573	453	482/457	Illumina Human610-Quad
	PGC	9394	12462		Several (see ref. 48)

BPD, bipolar disorder; SCZ, schizophrenia; NCNG, Norwegian Cognitive NeuroGenetics; TOP, Norwegian Thematically Organized Psychosis; WTCCC, British Wellcome Trust Case Control Consortium; Danish, Danish sub-sample of the Scandinavian Collaboration on Psychiatric Etiology; PGC, Psychiatric Genomics Consortium.

indicates the cases and controls in the single-centre samples that are also included in the PGC multi-centre sample.

Three independent GWASs for bipolar disorder were tested (Table 1): an extended BPD GWAS from the previously reported Norwegian TOP Study BPD sample [45], the German BPD GWAS [46], and the British Wellcome Trust Case Control Consortium (WTCCC) [47] BPD sample. We also tested the BPD multi-centre sample from the PGC [48]. 33% of the cases and 49% of the controls in the PGC sample overlap with the other samples tested (Table 1).

Thus, in this study, the PGC provides merged and extended samples rather than independent samples. This is potentially a more efficient and powerful approach for analyzing the data [49] than testing independent samples.

### Gene Scoring and Gene Set Enrichment Analysis

#### Scoring the Genes

In order to perform gene-based analyses on the GWASs, the single SNPs were first assigned to genes, by taking into account the physical position of the SNPs (i.e. within the boundaries of the genes) and the linkage disequilibrium (LD) of the genotyped SNPs with any other SNPs within the gene boundaries, based on the HapMap CEU reference sample [50]. This gene-based binning of SNPs was performed using the LDsnpR package [51] with the following parameters: the gene annotation used was the “Human Ensembl release 54” [52], the gene boundaries were set to 10kb upstream and downstream of the gene, the LD data used was the “HapMap Phase II release 27 in the CEU population”, and the pairwise LD threshold was set to r^2^ ≥0.8.

**Figure 1:**
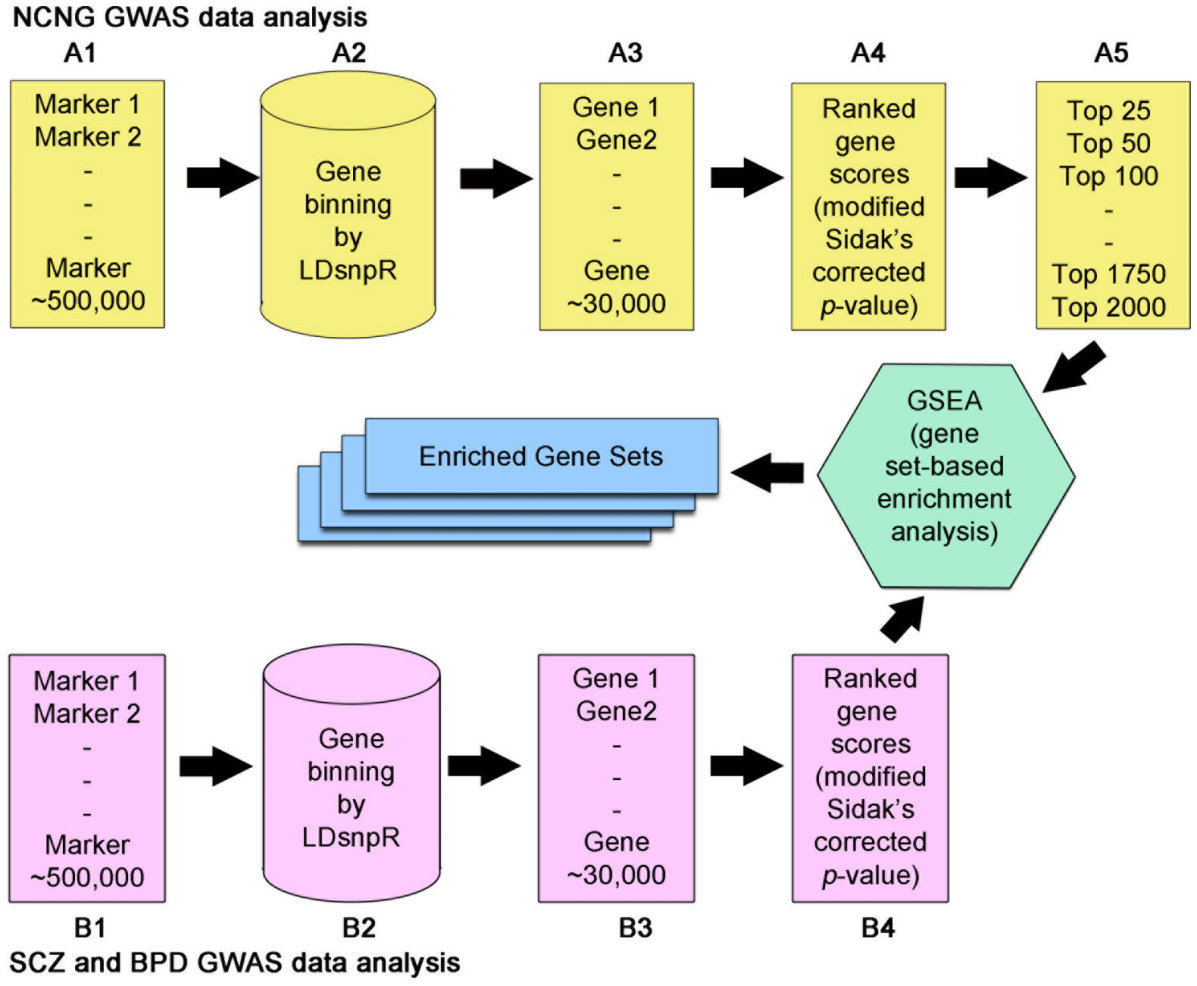
Schematic representation of the overall method. A1–A5: GWAS were performed for nine cognitive traits selected from the battery phenotyped in the healthy Norwegian NCNG sample (A1). Using the LDsnpR algorithm [51], SNPs were assigned to gene bins (A2–3) and the gene bins were scored using the minimum *p*-value corrected for the number of SNPs in the bin with an adjusted Sidak *p*-value. The gene scores were ranked (smallest Sidak *p*-value to biggest – A4). These GWAS-based ranked lists of genes were used to generate the candidate gene sets, which comprised the top 25, 50, 100, 250, 500, 750, 1000, 1250, 1500, 1750 and 2000 genes associated with each of the cognitive traits (A5). Thus, the candidate gene sets were overlapping, and there was an incremental increase in the number of genes per set. B1–B4: The GWAS data for the psychiatric disorders (B1) were subjected to the same pipeline for assigning SNPs to gene bins (B2–3), scoring (see manuscript), and ranking the genes by their score (smallest Sidak *p*-value to the biggest – B4).

After the SNPs were binned to genes, a gene-based association score was generated for each gene by assigning the minimal *p*-value from the SNPs in the bin, corrected for the number of SNPs in the bin with an adjusted Sidak's score [53], as implemented in LDsnpR [51], [54]. This method for adjusting the gene-based score for the number of SNPs has been shown previously to be comparable to permutation-based scores [55]. The gene scores were −log10 transformed and ranked (see Figure 1 for details of the overall procedure). All of the GWASs (NCNG, SCZ and BPD) were subjected to the same gene-based analytical protocol. The ranked gene lists from the NCNG GWASs were then used to produce candidate gene sets for the subsequent enrichment analysis in SCZ and BPD samples. For each of the nine neurocognitive traits, eleven gene sets were generated which contained the top 25, 50, 100, 250, 500, 750, 1,000, 1,250, 1,500, 1,750 and 2,000 most strongly associated genes (i.e. 99 cognitive trait-associated gene sets in total). Sets of genes of different sizes were selected in order to represent different significance thresholds, independent of *p*-value thresholds which are influenced by the power of particular individual GWAS and therefore not directly comparable across GWASs. By scanning different thresholds, we aimed to identify the set of top genes with the strongest evidence of enrichment. The International Schizophrenia Consortium has used similar multiple-threshold approaches when analysing GWAS data to determine the amount of variance that can be explained by including variants of lower effect size [56].

#### Gene Set Enrichment Analyses

The Gene Set Enrichment Analysis (GSEA) method has been developed and extensively used for global gene expression studies, but is increasingly being used in the analysis of GWAS [54], [57]–[59]. It assesses whether a predefined gene set shows significant enrichment of signal (or association in GWAS) in a ranked list of genes, i.e. if the selected set clusters at the top of the ranked lists or is randomly distributed throughout the list. Here we used GSEA to test the cognitive trait-associated gene sets for enrichment of association in the ranked genes from GWASs of psychiatric disorders (Figure 1).

GSEA calculates an enrichment score (ES) that increases when a gene from the (neurocognitive) gene set is identified among the ranked list of genes emerging from the (psychiatric) GWAS and decreases when it is not. Specifically, we used a weighted (p = 1) ES, which weights the genes within the gene set by the strength of their association with the phenotype and thus assigns a higher ES to gene sets clustering higher up in the ranked list as opposed to those clustering in the middle of the ranked list. The significance, or *p*-value, of the ES is determined by permuting (1000 times) the ranked list and recalculating the ES to create a null distribution to which the ES of the candidate gene set is compared. Each GSEA was performed three times in order to ensure the reproducibility of the permutation-based *p*-values. In addition, GSEA produces a normalized ES (NES), which is based on the ESs for all dataset permutations and enables comparison of NESs across gene sets. Here, all 99 cognitive trait-associated gene sets were tested together using the gene matrix file format option in GSEA in order to utilize and retain the false discovery rate (FDR) *q*-value. The FDR is an estimate of the proportion of significant results (at *p*≤0.05) that are false positives and has become widely accepted as a powerful multiple-testing correction approach in GWAS [60]. As implemented in GSEA, the FDR *q*-value adjusts both for the multiple (i.e. 99) gene sets tested simultaneously and for the gene set size [61]. The average FDR *q*-value of the three runs is reported, together with the corresponding *p*-values. Gene sets with a *p*-value≤0.05 and an FDR *q*-value≤0.25 were declared nominally significantly enriched and taken forward for subsequent validation, as described below.

We performed additional validation tests in order to evaluate the robustness of our findings. Detailed methods are presented in the Supporting Information. Notably, we used a random sets approach to validate the findings. For each of the nominally enriched sets (i.e. *p*-value≤0.05 and FDR *q*-value≤0.25), we generated 100 random gene sets that mimicked the candidate gene sets with respect to the number of genes in the set, the number of SNPs within each gene, and the total number of sets tested together. These 100 mimic sets were run together with the candidate sets in other to determine whether the enrichment was higher in the candidate sets than the random sets. Candidate gene sets which scored an NES higher than 98% of the random sets was declared statistically significantly enriched.

## Results

From the battery of cognitive tests used to phenotype a sample of 670 healthy Norwegians [27], we selected 9 cognitive traits relevant to SCZ and BPD. General cognition/intelligence was assessed by the “Estimated IQ”, the “Matrix Reasoning” and the “Verbal Abilities” from the Wechsler abbreviated scale of intelligence [35]; verbal memory by “Learning” and “Delayed Verbal Memory” from the California Verbal Learning Test [37]; executive function by the 3^rd^ condition (“Colour-word interference) of the Delis-Kaplan Executive function system colour-word interference test and visuospatial attention by three reaction times obtained during the Cued Discrimination Task (the valid, invalid and neutral conditions [38], “Visuospatial attention 1/2/3”). Further details on the full battery of tests carried out on the NCNG sample are given in Table S1 in File S1 and in Espeseth *et al.* [27]. The correlations between the selected traits at the phenotypic and gene levels are given in Tables S2 and S3 in File S1, respectively. For each of the 9 traits, we performed a gene-based analysis of the GWAS, constructing candidate gene sets comprising the most associated genes. These gene sets were then tested for enrichment of association in previously published, independent GWASs of SCZ [42]–[44] and BPD [45]–[47]. The workflow is shown in Figure 1.

The enrichment of association was tested using Gene Set Enrichment Analysis (GSEA). The multiple-testing correction FDR *q*-value was used to correct for gene set size and the number of gene sets tested (see the Supporting Information for explanation of the different values). In expression studies, a nominal threshold of significance (*p*-value≤0.05) and an FDR *q*-value≤0.25) is recommended [61]. For GWAS, an optimal FDR threshold has not yet been established. Thus, an FDR *q*-value≤0.25 was retained as an initial threshold in order to select gene sets for further validation. Detailed methods are presented in the Supporting Information. Furthermore, the *q-*value corrects for the number of genes sets tested (99 in this study) based on the assumption that these sets are independent. In this study, however, the sets are not independent since the 11 sets generated for each trait are nested and the traits themselves are correlated (Table S3 in File S1). Thus the FDR *q*-value correction may be considered conservative.

In particular, for each of the enriched sets (i.e. *q*-value≦0.25 and *p*-value≦0.05) in each of the psychiatric disorder samples tested, we generated 100 random gene sets that mimicked the candidate gene sets with respect to the number of genes in the set, the number of SNPs within each gene, and the total number of sets tested together. Candidate gene sets which showed stronger evidence of enrichment, as determined by the NES, than greater than 98% of the random gene sets were considered validated. (measured by the normalized enrichment score, see Supporting Information).

As a means of further validation, we repeated the gene set-based analyses using a set of housekeeping genes (Table S5 in File S1), and we also tested the cognitive gene sets against the WTCCC non-psychiatric datasets in order to demonstrate specificity to psychiatric phenotypes. The lowest *q*-value obtained was 0.21, for Type 2 Diabetes (Table S6 in File S1). The final validation test, which involved pruning genes that were in high LD with other genes in the list, was designed to ensure that the observed enrichment of the neurocognitive gene sets in the SCZ datasets is not entirely due to LD between genes (Table S7 in File S1).

For BPD, the most significantly enriched gene set was the visuospatial attention.1 −25 set in the German sample. Another visuospatial attention set (visuospatial attention.2 −25) also showed enrichment in the German sample and a trend towards enrichment in the WTCCC sample. The difference between these two tests is that the valid condition is analyzed in visuospatial attention.1 while the invalid condition is analyzed in visuospatial attention.2 [38]. At the cognitive level, these are two different tests, even though they are highly correlated (0.97, Table S2 in File S1); at the gene level the correlation was smaller (0.50, Table S3 in File S1). In addition, several of the sets for verbal abilities in the German sample (−25, −50, and −100) and in the WTCCC samples (−25) met our pre-defined criteria for enrichment and were validated; the delayed verbal memory-25 set also showed enrichment in the German sample (see Table 2 for the *p*- and *q*-values and Table S4 in File S1 for the mimic set test results). None of these sets showed significant enrichment in either the smaller TOP sample or the PGC sample, but, notably, sets for verbal abilities, visuospatial attention and delayed verbal memory had the lowest FDR *q-*values in these samples.

**Table 2:** Testing gene sets associated with normal neurocognitive variation for enrichment of association with bipolar disorder.

Sample	German			WTCCC			Norwegian - TOP			PGC		
Cases/controls	682/1300			1868/2938			575/417			7481/9250		
Gene sets	R	*p*-value	*q*-value	R	*p*-value	*q*-value	R	*p*-value	*q*-value	R	*p*-value	*q*-value
**Visuospatial attention.1 −25**	1^a^	0.00*	0.0063	n.e.	-	-	n.e.	-	-	n.e.	-	-
**Visuospatial attention.2 −25**	2^a^	0.0047	0.023	4^b^	0.14	0.27	n.e.	-	-	n.e.	-	-
**Verbal abilities −25**	3^a^	0.0073	0.033	2^a^	0.0047	0.036	n.e.	-	-	n.e.	-	-
**Verbal abilities −50**	4^a^	0.0063	0.073	n.e.	-	-	n.e.	-	-	1^b^	0.02	0.42
**Verbal abilities −100**	5^a^	0.0013	0.088	n.e.	-	-	1^b^	0.19	0.28	n.e.	-	-
**Visuospatial attention.3 −25**	6	0.04	0.096	n.e.	-	-	n.e.	-	-	n.e.	-	-
**Visuospatial attention.3 −50**	9	0.028	0.12	n.e.	-	-	n.e.	-	-	n.e.	-	-
**Learning −100**	10	0.013	0.17	n.e.	-	-	n.e.	-	-	n.e.	-	-
**Delayed verbal memory −100**	11	0.037	0.17	n.e.	-	-	n.e.	-	-	2^b^	0.042	0.54
**Verbal abilities −250**	12	0.0007	0.18	n.e.	-	-	n.e.	-	-	5^b^	0.016	0.63
**Learning −250**	13	0.002	0.21	n.e.	-	-	n.e.	-	-	n.e.	-	-
**Verbal abilities −500**	14	0.00*	0.22	n.e.	-	-	n.e.	-	-	n.e.	-	-
**Delayed verbal memory −50**	n.e.	-	-	3^b^	0.063	0.26	n.e.	-	-	n.e.	-	-
**Delayed verbal memory −25**	n.e.	-	-	1^a^	0.0037	0.028	n.e.	-	-	n.e.	-	-
**Colour-word interference −25**	n.e.	-	-	n.e.	-	-	n.e.	-	-	4^b^	0.23	0.62
**Matrix reasoning −100**	n.e.	-	-	n.e.	-	-	5^b^	0.22	0.29	n.e.	-	-
**Delayed verbal memory −1000**	n.e.	-	-	5^b^	0.041	0.29	4^b^	0.0073	0.29	n.e.	-	-
**Visuospatial attention.1 −2000**	n.e.	-	-	n.e.	-	-	2^b^	0.00033	0.28	n.e.	-	-
**Colour-word interference −50**	n.e.	-	-	n.e.	-	-	n.e.	-	-	3^b^	0.095	0.58
**Visuospatial attention.1 −500**	n.e.	-	-	n.e.	-	-	3^b^	0.033	0.29	n.e.	-	-

*q*-value, obtained from 3 GSEA runs with 1,000 permutations each). The maximum standard deviation from the average *q*-value was 0.07. Sets that passed the enrichment threshold (*p*-value≤0.05, FDR *q*-value≤0.25) were tested for validation using random mimic sets (see Table S4 in File S1). For each GWAS dataset, the 5 most enriched candidate sets are shown. For the German dataset, the 14 most enriched sets are presented to show the overlap with the other datasets. The rank position (R) of the gene set within the total number of gene sets tested is determined by the average false discovery rate (

% of the random sets (i.e. validated sets).^a^ indicates sets that were more enriched than 98

^b^ indicates sets that did not pass the enrichment threshold but were among the 5 most enriched in the corresponding sample.

“n.e.”. Visuospatial attention.1 – Visuospatial attention task with valid cue to the location of the visual target; Visuospatial attention.3 – Visuospatial attention task with neutral cue to the location of the visual target. The number after each gene set name represents the number of genes within that set (e.g. the Colour-word interference −25 set contains the top 25 genes within the colour-word interference ranking list of genes). Sets that did not pass the enrichment threshold and ranked outside the top 5 are indicated by

*p-*value of zero (0.0) indicates an actual *p*-value of less than 1/number-of-permutations. A reported

Again using the same criteria to define enriched sets, we observed that for SCZ, the colour-word interference −25 gene set was enriched both in the German-Dutch sample and in the Danish sample (see Table 3 for the *p*- and *q*-values and Table S4 in File S1 for the mimic set test results). The colour interference −50 set also showed significant enrichment in the Danish sample, but the enrichment was borderline in the German-Dutch sample. The learning −250 set was enriched in the PGC sample. The *q*-value can be sensitive to sample-specific factors, like the size of the sample, which may be relevant to smaller samples like the Norwegian TOP sample, or the heterogeneity of the sample, which is most likely to be relevant to multi-centre samples such as the PGC samples. While only one gene set met our criteria for significant enrichment and subsequent validation in the PGC, the five most enriched sets are provided. Interestingly, although the colour interference −25 set did not surpass the significance threshold in the Norwegian-TOP and PGC samples, it was the 1^st^ and 2^nd^ most strongly enriched set in these samples respectively. The visuospatial sets also showed a trend for enrichment in these two samples.

**Table 3:** Testing gene sets associated with normal neurocognitive variation for enrichment of association with schizophrenia.

Sample	German-Dutch			Danish			Norwegian			PGC		
Cases/controls	1169/3714			573/453			405/417			9394/12464		
Gene set	R	*p*-value	*q*-value	R	*p*-value	*q*-value	R	*p*-value	*q*-value	R	*p*-value	*q*-value
**Colour-word interference −25**	1^a^	0.011	0.14	2^a^	0.0053	0.081	1^b^	0.061	0.42	2^b^	0.086	0.34
**Estimated IQ −25**	2	0.023	0.14	n.e.	-	-	n.e.	-	-	n.e.	-	-
**Colour-word interference −50**	3^b^	0.021	0.29	1^a^	0.002	0.077	n.e.	-	-	n.e.	-	-
**Matrix reasoning −100**	4^b^	0.012	0.39	n.e.	-	-	n.e.	-	-	n.e.	-	-
**Matrix reasoning −250**	5^b^	0.001	0.4	n.e.	-	-	n.e.	-	-	n.e.	-	-
**Visuospatial attention.1 −25**	n.e.	-	-	4^b^	0.26	0.24	n.e.	-	-	3^b^	0.05	0.35
**Visuospatial attention.3 −25**	n.e.	-	-	n.e.	-	-	2^b^	0.14	0.6	n.e.	-	-
**Visuospatial attention.3 −50**	n.e.	-	-	n.e.	-	-	3^b^	0.13	0.61	n.e.	-	-
**Visuospatial attention.3 −1500**	n.e.	-	-	n.e.	-	-	4^b^	0.13	0.83	n.e.	-	-
**Visuospatial attention.3 −1000**	n.e.	-	-	n.e.	-	-	5^b^	0.18	0.84	n.e.	-	-
**Verbal abilities −25**	n.e.	-	-	3^b^	0.26	0.23	n.e.	-	-	n.e.	-	-
**Learning −500**	n.e.	-	-	5^a^	0.01	0.24	n.e.	-	-	4^b^	0.00*	0.48
**Learning −250**	n.e.	-	-	n.e.	-	-	n.e.	-	-	1^a^	0.00*	0.13
**Verbal abilities −750**	n.e.	-	-	n.e.	-	-	n.e.	-	-	5^b^	0.12	0.53

*q*-value, obtained from 3 GSEA runs with 1,000 permutations each). The maximum standard deviation from the average *q*-value was 0.06. Sets that passed the enrichment threshold (*p*-value≤0.05, FDR *q*-value≤0.25) were tested for validation using random mimic sets (see Table S4 in File S1). For each GWAS dataset the 5 most enriched candidate sets are shown. The rank position (R) of the gene set within the total number of gene sets tested was determined by the average false discovery rate (

% of the random sets (i.e. validated sets).^a^ indicates sets that were more enriched than 98

^b^ indicates sets that did not pass the enrichment threshold but were among the 5 most enriched in the corresponding sample.

“n.e.”. Visuospatial attention.1 – Visuospatial attention task with valid cue to the location of the visual target; Visuospatial attention.3 – Visuospatial attention task with neutral cue to the location of the visual target. The number after each gene set name represents the number of genes within that set (e.g. the Colour-word interference −25 set contains the top 25 genes within the colour-word interference ranking list of genes). Sets that did not pass the enrichment threshold and ranked outside the top 5 are indicated by

Interestingly, the enrichment signal in each of the SCZ and BPD GWASs was driven by different genes within the colour-word interference candidate set for SCZ (see Table S8 in File S1) and within the verbal abilities, visuospatial attention and delayed verbal memory sets for BPD (see Table S9 in File S1), attesting to the heterogeneity and polygenicity of these traits, and to the utility of such integrated approaches.

In addition, considering the hypothesis that SCZ and BPD overlap at the genetic level, we looked for overlap of the most strongly enriched cognitive gene sets in the PGC SCZ and BPD samples, as they are the biggest samples tested (Table S10 in File S1). Considering the enrichment rank, several gene sets for colour interference, verbal abilities and visuospatial attention were among the top 10 most enriched sets for both disorders, but no candidate set was significantly enriched in both disorders (at *q*-value≤0.25).

## Discussion

In this study we identified candidate sets of genes associated with cognitive abilities in healthy adults, and we screened these sets for specific enrichment of association in SCZ or BPD. We chose to perform gene-based analysis as this method allows for more flexibility in the comparison of genetic effects between traits than single-marker methods. In particular, and in contrast to standard single-marker analysis, gene-based analyses allow for allelic heterogeneity [24], [51], [62]. The results of our study provide evidence that cognitive abilities might be suitable phenotypes to use for the identification of genetic factors overlapping with those implicated in psychiatric disorders.

Gene sets associated with verbal abilities, visuospatial attention and delayed verbal memory were the most enriched for association in the BPD datasets. Neurocognitive dysfunctions are not as well defined in BPD as they are in SCZ [28], [63], [64]. For SCZ, deficits in executive function, verbal abilities (learning and memory), attention and speed of processing [29]–[31] have been reported, which corroborate our findings. It has been observed that the cognitive functions impaired in SCZ are often also impaired in BPD but to a lesser extent, and that cognitive dysfunction is determined more by history of psychosis than by standard diagnosis [32]. From this perspective, it would be interesting to apply the approach we describe here to cross-disorder samples, comprising patients who share common clinical symptoms, such as psychosis for instance. In the extended PGC BPD sample, none of the enrichments were significant, which might reflect a higher clinical or genetic heterogeneity between and/or within samples for BPD than for SCZ. In other genetic studies, it has often been observed that the genetic signal is stronger for SCZ than for BPD [65]. These problems could be overcome by analyzing even better annotated BPD samples to gain power in more homogenous samples. Further work is needed to establish whether particular subgroups of BPD patients show greater impairment in neurocognitive control and to determine whether the enrichment of association of the different gene sets is more consistent in specific subgroups.

For SCZ, gene sets associated with the colour-word interference test show the greatest enrichment out of all the sets analyzed. Cognitive neuroscience studies of SCZ are pointing towards deficits in information-processing functions involved in cognitive control or executive functions – the ability to regulate, coordinate, and sequence thoughts and actions in accordance with internally maintained behavioral goals [33], [66]. Performance in colour-word interference tests partly depends on cognitive inhibition, a set of processes that play important roles in cognitive control. Deficits in neurocognitive tests of cognitive control are observed in patients with SCZ. Specifically in the colour-word interference test, patients with SCZ tend to use more time in the interference condition than controls, indicating impaired cognitive inhibition [67]. Such cognitive inhibition, a subdomain of cognitive control that is especially engaged during the colour-word interference test, involves “top-down” regulation from the prefrontal cortex areas to subcortical areas, especially from the dorsolateral prefrontal and the anterior cingulate cortices, which have also been recurrently implicated in SCZ [68], [69]. At the genetic level, it has been shown that individual loci implicated in SCZ can also influence cognitive control. For example the variant rs1344706 in the *ZNF804A* gene, which is well characterized for its effect in SCZ [70], is associated with variability in activation of prefrontal cortex areas during a cognitive control task [71]. Here, using a gene-based polygenic approach and several large, independent samples, we provide evidence of an overlap between the group of genes that influence colour-word interference in healthy adults and genes associated with SCZ.

The learning −250 gene set was the most significantly enriched in the large PGC SCZ GWAS, and the learning −500 set was enriched in the Danish sample. These sets contained genes that are associated with the learning slope during a memory test (the California Verbal Learning Test, [36]). Impairments in memory in general have been reported in patients with SCZ, though these impairments have been observed for both episodic and working memory [34]. Here the delayed verbal memory sets did not show enrichment in the samples that were tested, but this could be due to a lack of power. Despite this negative result, it is interesting to note that a genetic approach can be used to dissect the overlap between cognitive functions and SCZ.

While our results require replication, further validation and extension to other cognitive traits and other psychiatric disorders, it is encouraging to observe that genetic studies using polygenic and pleiotropic models are converging with other approaches in implicating specific cognitive functions in psychiatric disorders. Additional gene set-based studies might help to elucidate and dissect the relationship between cognitive functions and dysfunctions. Several cognitive functions implicated by our study will need further investigation, especially the functions recruited during the colour interference test, which has not gained as much support as a potential endophenotype for SCZ as other traits. We also highlight the need for samples with higher levels of phenotypic characterization in order to better deconstruct the effect of cognitive impairments in psychiatric disorders at the genetic level.

## Supporting Information

File S1 This file contains ten supporting tables (S1–S10) and supporting methods.
(DOCX)
