# Supplementary material for: A Genetic Deconstruction of Neurocognitive Traits in Schizophrenia and Bipolar Disorder
Source: PLoS One. 2013 Dec 12;8(12):e81052. doi: 10.1371/journal.pone.0081052 (PMC3861303; doi:10.1371/journal.pone.0081052)
Supplement: File S1 — This file contains ten supporting tables (S1–S10) and supporting methods. (DOCX) [file pone.0081052.s001.docx]

**FILE S1: SUPPORTING INFORMATION**

**A genetic deconstruction of neurocognitive traits in schizophrenia and bipolar disorder**

#### **Table S1.** **Main NCNG cognitive tests and respective cognitive functions** (adapted from [1]). The wide-ranging battery of cognitive tests is shown, together with the cognitive functions they cover. Functions tested in this study are denoted by * and their short names, as used in the tables and the manuscript, are specified.

| **Cognitive test** | **Cognitive function** | **References** |
| --- | --- | --- |
| Wechsler Abbreviated Scale of Intelligence Vocabulary | Verbal Abilities* | Wechsler, 1999 [2] |
| Wechsler Abbreviated Scale of Intelligence Matrix reasoning | Matrix Reasoning* | Wechsler, 1999 [2] |
| Wechsler Abbreviated Scale of Intelligence | Estimated IQ* | Wechsler, 1999 [2] |
| California Verbal Learning Test: Learning* and Delayed verbal memory* | Verbal memory | Delis *et al*., 2000 [3] |
| Delis-Kaplan Executive Function System, Colour-Word Interference* Test | Executive attention | Delis *et al*., 2001[4] |
| Letter-number span | Working memory | Delis *et al.* (2001) [4] |
| Cue Discrimination Task, valid (1), invalid (2) and neutral (3) conditions | Visuospatial attention* | Parasuraman *et al.* (1992) [5] |
| Trail-making test A & B | Psychomotor speed/ executive attention | Spreen & Strauss (1998) [6] |
| Digit-symbol substitution | Psychomotor speed/ executive attention | Delis *et al*., 2001 [4] |
| Mini-mental State Examination | General cognitive function | Folstein *et al*., 1975 [7] |
| Beck Depression Inventory | Depression | Beck *et al*., 1961 [8] |
| Life event interview | General |  |

#### **Table S2. Correlation between NCNG neurocognitive test results.**

|  | **Verbal abilities** | **Matrix Reasoning** | **Estimated IQ** | **Colour-word Interference** | **Learning** | **Delayed Verbal Memory** | **Visuospatial attention.1** | **Visuospatial attention.2** | **Visuospatial attention.3** |
| --- | --- | --- | --- | --- | --- | --- | --- | --- | --- |
| **Verbal abilities** | 1 |  |  |  |  |  |  |  |  |
| **Matrix Reasoning** | 0.25 | 1 |  |  |  |  |  |  |  |
| **Estimated IQ** | 0.80 | 0.64 | 1 |  |  |  |  |  |  |
| **Colour-word Interference** | -0.11 | -0.42 | -0.16 | 1 |  |  |  |  |  |
| **Learning** | 0.30 | 0.40 | 0.31 | -0.36 | 1 |  |  |  |  |
| **Delayed Verbal Memory** | 0.28 | 0.35 | 0.27 | -0.30 | 0.81 | 1 |  |  |  |
| **Visuospatial attention.1** | -0.17 | -0.42 | -0.20 | 0.51 | -0.36 | -0,.3 | 1 |  |  |
| **Visuospatial attention.2** | -0.08 | -0.40 | -0.12 | 0.51 | -0.35 | -0.30 | 0.97 | 1 |  |
| **Visuospatial attention.3** | -0.08 | -0.43 | -0.14 | 0.52 | -0.36 | -0.32 | 0.98 | 0.98 | 1 |


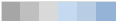


Pearson’s correlation coefficient (r)

-1

1

#### **Table S3.** **Representation of the overlap between the NCNG gene sets containing 25 and 50 genes**

|  | **Verbal Ab.-25** | **Verbal Ab.-50** | **Matrix reasoning-25** | **Matrix reasoning-50** | **Estimated IQ-25** | **Estimated IQ -50** | **Colour-word Interf-25** | **Colour-word Interf--50** | **Learn. -25** | **Learn-50** | **Delayed Verb. Memo-25** | **Delayed Verb. Memo-50** | **Visuosp. Att.1-25** | **Visuosp. Att.1-50** | **Visuosp. Att.2-25** | **Visuosp. Att.2-50** | **Visuosp. Att.3-25** | **Visuosp. Att.3-50** |
| --- | --- | --- | --- | --- | --- | --- | --- | --- | --- | --- | --- | --- | --- | --- | --- | --- | --- | --- |
| **Verbal Ab.-25** | 1 |  |  |  |  |  |  |  |  |  |  |  |  |  |  |  |  |  |
| **Verbal Ab.-50** | 0.50 | 1 |  |  |  |  |  |  |  |  |  |  |  |  |  |  |  |  |
| **Matrix reasoning-25** | 0 | 0 | 1 |  |  |  |  |  |  |  |  |  |  |  |  |  |  |  |
| **Matrix reasoning-50** | 0 | 0 | 0.50 | 1 |  |  |  |  |  |  |  |  |  |  |  |  |  |  |
| **Estimated IQ -25** | 0.10 | 0.10 | 0.14 | 0.14 | 1 |  |  |  |  |  |  |  |  |  |  |  |  |  |
| **Estimated IQ -50** | 0.14 | 0.16 | 0.16 | 0.18 | 0.50 | 1 |  |  |  |  |  |  |  |  |  |  |  |  |
| **Colour-word Interf.-25** | 0 | 0 | 0 | 0 | 0 | 0 | 1 |  |  |  |  |  |  |  |  |  |  |  |
| **Colour-word Interf -50** | 0 | 0 | 0 | 0 | 0 | 0 | 0.50 | 1 |  |  |  |  |  |  |  |  |  |  |
| **Learn.-25** | 0 | 0 | 0 | 0 | 0 | 0 | 0 | 0 | 1 |  |  |  |  |  |  |  |  |  |
| **Learn.—50** | 0 | 0 | 0 | 0 | 0 | 0 | 0 | 0 | 0.50 | 1 |  |  |  |  |  |  |  |  |
| **Delayed Verb. Memo-25** | 0 | 0 | 0 | 0 | 0 | 0 | 0 | 0 | 0.06 | 0.10 | 1 |  |  |  |  |  |  |  |
| **Delayed Verb. Memo-50** | 0 | 0 | 0 | 0 | 0 | 0 | 0 | 0 | 0.12 | 0.20 | 0.50 | 1 |  |  |  |  |  |  |
| **Visuosp. Att.1-25** | 0 | 0 | 0 | 0 | 0 | 0 | 0 | 0 | 0 | 0 | 0 | 0 | 1 |  |  |  |  |  |
| **Visuosp. Att.1-50** | 0 | 0 | 0 | 0 | 0 | 0 | 0 | 0 | 0 | 0 | 0 | 0 | 0.50 | 1 |  |  |  |  |
| **Visuosp. Att.2-25** | 0 | 0 | 0 | 0 | 0 | 0 | 0 | 0 | 0 | 0 | 0 | 0 | 0.24 | 0.42 | 1 |  |  |  |
| **Visuosp. Att.2-50** | 0 | 0 | 0 | 0 | 0 | 0 | 0 | 0 | 0 | 0 | 0 | 0 | 0.36 | 0.72 | 0.50 | 1 |  |  |
| **Visuosp. Att.3-25** | 0 | 0 | 0 | 0 | 0 | 0 | 0 | 0 | 0 | 0 | 0 | 0 | 0.28 | 0.50 | 0.30 | 0.40 | 1 |  |
| **Visuosp. Att.3-50** | 0 | 0 | 0 | 0 | 0 | 0 | 0 | 0 | 0 | 0 | 0 | 0 | 0.46 | 0.82 | 0.44 | 0.74 | 0.01 | 1 |


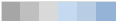
Proportion of shared genes: Low High

Verbal Ab. - verbal abilities, Colour-word Interf – colour-word interference, Learn - learning, Delayed Verb. Memo - delayed verbal memory, Visuosp. Att. - visuospatial attention.

**SUPPORTING INFORMATION METHODS**

**Validation Tests**

A generally accepted FDR q-value threshold for gene expression studies is 0.25 [9]. However, as GSEA has only recently been applied to GWAS data, it is not clear whether this threshold is equally acceptable for this purpose. Thus, to exclude spurious, non-specific or biased results, we performed four additional validation tests to establish the significance of our finding.

1. Mimic Gene Sets

For each of the significantly enriched gene sets in each of the GWASs tested (FDR *q*-value ≤ 0.25 and *p*-value ≤ 0.05), we produced 100 random gene sets that mimic the candidate gene sets with respect to the number of genes in the sets and the number of SNPs binned to each gene. These gene sets were selected randomly from the whole genome as described previously by Ersland *et al*. [10]. The 100 random gene sets were then run together using the same GSEA procedure that generated the original gene sets. Since we wanted to test whether our candidate sets were more enriched than random similar sets of genes, we here report the normalized enrichment score (NES) for each candidate set, and how each candidate set ranked in the 3 runs.

**Table S4. Comparison of normalized enrichment score for the candidate sets enriched with 100 mimic sets.** The candidate sets that were more enriched then 98% of the mimic sets are highlighted in green.

| **Bipolar disorder sample origin** | |  | **Ranks** |  |  |
| --- | --- | --- | --- | --- | --- |
|  | **Gene sets** | **Average NES** | **Run 1** | **Run 2** | **Run 3** |
| **German** | Visuospatial attention.1 -25 | 1.54 | 1 | 1 | 1 |
|  | Visuospatial attention.2 -25 | 1.45 | 2 | 3 | 1 |
|  | Verbal abilities -25 | 1.42 | 1 | 2 | 1 |
|  | Verbal abilities -50 | 1.35 | 2 | 2 | 2 |
|  | Verbal abilities -100 | 1.31 | 1 | 1 | 1 |
|  | Verbal abilities -250 | 1.21 | 1 | 1 | 1 |
|  | Visuospatial attention.3 -25 | 1.31 | 3 | 4 | 5 |
|  | Visuospatial attention.3 -50 | 1.26 | 5 | 5 | 5 |
|  | Learning -100 | 1.20 | 5 | 5 | 5 |
|  | Learning -250 | 1.42 | 1 | 1 | 1 |
|  | Verbal abilities -500 | 1.17 | 2 | 2 | 2 |
|  | Delayed verbal memory -100 | 1.18 | 6 | 7 | 7 |
| **WTCCC** | Delayed verbal memory -25 | 1.46 | 1 | 1 | 1 |
|  | Verbal abilities -25 | 1.46 | 1 | 1 | 1 |
| **Schizophrenia sample origin** | |  |  |  |  |
|  | **Gene sets** | **Average NES** | **1st run** | **2nd run** | **3rd run** |
|  | Colour-word interference -25 | 1.39 | 1 | 2 | 1 |
| **German-Dutch** | Estimated IQ -25 | 1.34 | 3 | 3 | 3 |
| **Danish** | Colour-word interference -25 | 1.40 | 1 | 1 | 1 |
|  | Colour-word interference -50 | 1.35 | 2 | 2 | 2 |
|  | Learning -500 | 1.11 | 1 | 1 | 1 |
| **PGC** | Learning -250 | 1.36 | 1 | 1 | 1 |

2. Housekeeping Genes Dataset

A gene set containing 37 “housekeeping genes” was tested against the SCZ GWAS datasets. Housekeeping genes show stable expression across different human tissues and encode proteins that are involved in cell function and maintenance. Such genes could, therefore, be used as a suitable control gene set in this study. The gene set was selected from the Applied Biosystems list of TaqMan endogenous controls and from Warrington *et al.* ([11]; see Ersland *et al.* [10] for more details). The association significance of the housekeeping gene set was assessed from the average FDR *q*-value obtained from 3 GSEA runs with 1,000 permutations each (Table S4).

**Table S5. GSEA results for the housekeeping gene set against the genes from the SCZ GWASs.** The average FDR *q*-values for the housekeeping gene set in each SCZ GWAS are shown.

|  |  | **Housekeeping genes** |
| --- | --- | --- |
| **SCZ** | **German-Dutch** | 0.33 |
|  | **Danish** | 0.56 |
|  | **Norwegian TOP** | 0.22 |
|  | **PGC** | 0.88 |

3. Wellcome Trust Non-Psychiatric GWAS Datasets

The candidate gene sets were tested against the Wellcome Trust Case Control Consortium (WTCCC) non-psychiatric GWAS datasets [12], which comprise Crohn´s disease, coronary artery disease, rheumatoid arthritis, hypertension, type 1 diabetes and type 2 diabetes. The association significance of the neurocognitive gene sets with the non-psychiatric datasets was determined by the average FDR *q*-value obtained from 3 GSEA runs with 1,000 permutations each (Table S5). The results show that overall the *q*-values obtained were higher than the *q*-values obtained for the top neurocognitive gene sets (i.e. colour-word interference) in the SCZ datasets. The minimum *q*-value obtained in WTCCC datasets was 0.21 (for HT), while the colour-word interference -25 gene set in SCZ showed *q*-values lower than this (0.142 and 0.081) in two of the datasets (German-Dutch and Danish). Furthermore, overall, the *q*-values for the most significant colour-word interference gene sets in the SCZ datasets are lower than those in the WTCCC non-psychiatric datasets.

**Table S6. GSEA results of the candidate gene sets against the WTCCC non-psychiatric GWASs.** The lowest average FDR *q*-values observed for the 99 candidate gene sets are shown, together with the *q*-values for the colour-word interference -25 and -50 gene sets.

|  | **Lowest *q*-value observed for 99 candidate sets** | ***q-*value colour-word interference -25** | ***q*-value colour-word interference -50** |
| --- | --- | --- | --- |
| **Coronary Artery Disease** | 0.89 | 1.00 | 0.89 |
| **Crohn’s Disease** | 0.50 | 1.00 | 1.00 |
| **Hypertension** | 0.21 | 0.99 | 1.00 |
| **Rheumatoid Arthritis** | 0.60 | 1.00 | 1.00 |
| **Type 1 Diabetes** | 0.58 | 1.00 | 0.99 |
| **Type 2 Diabetes** | 0.21 | 0.25 | 0.98 |

4. Curated Gene Set

We performed a test to show that the signal coming from the different genes within each gene set was not biased by the LD-based clustering of the genes. The most significantly enriched gene set in the SCZ dataset (colour-word interference -25) was manually curated, or pruned, to ensure that no two genes in the list were selected due to being assigned the same association signal because of their proximity or LD. One hundred random gene sets mimicking this pruned gene set (with respect to the number of genes and the number of SNPs within the genes) were also generated. These 101 gene sets (100 random sets plus the actual pruned set) were tested for enrichment against the SCZ GWASs using GSEA (using the protocol described in the main text) and the number of random sets that produced a better *q*-value than the actual pruned set was determined (Table S6).

**Table S7. Number of random gene sets that had a *q*-value lower than the manually curated colour-word interference -25 set after GSEA of the SCZ GWASs**

| **SCZ GWAS** | **No. of random sets with *q*-value lower than pruned set** |
| --- | --- |
| German-Dutch | 2.3 |
| Danish | 8.7 |
| Norwegian TOP | 38.0 |
| PGC | 8.0 |

This method should be considered conservative because while the gene sets are manually pruned, the ranked GWAS-based gene list is not. Thus, the score of the gene set is penalized for not containing a gene ranked high in the gene list that is representing the same genetic signal as the gene that survived the manual curation. Using this conservative approach, the pruned candidate set scores within the top 10 on the enrichment score list, with the exception of the Norwegian TOP dataset which has the lowest sample size and thus possibly the least power. Therefore, the observed enrichment of the neurocognitive gene sets in the SCZ datasets is not entirely due to possible LD between genes.

### FDR *q*-value and nominal *p*-value considerations.

Here we copy explanations from the GSEA User Guide web page (http://www.broadinstitute.org/gsea/doc/GSEAUserGuideFrame.html) to guide the interpretation of FDR *q*-values versus *p*-values. We have directly quoted the original text as the explanation is as clear as it can be.

*“The nominal p value estimates the statistical significance of the enrichment score for a single gene set. However, when you are evaluating multiple gene sets, you must correct for gene set size and multiple hypothesis testing. Because the p value is not adjusted for either, it is of limited value when comparing gene sets. The Gene Set Enrichment Analysis PNAS paper* [13] *describes the p value statistic in the section titled Appendix: Mathematical Description of Methods.*

*The FDR is adjusted for gene set size and multiple hypotheses testing while the p value is not. When a top gene set has a small nominal p value and a high FDR value, it generally indicates that it is not as significant when compared with other gene sets in the empirical null distribution. This could be because you do not have enough samples, the biological signal is subtle, or the gene sets do not represent the biology in question very well. On the other hand, the FDR is based on two distributions of all gene sets; if only one of many gene sets is enriched, that gene set is likely to have a high FDR. Finally, a top gene set with a high nominal p value and a low FDR value, generally indicates a negative result: the gene set itself is not significant and other sets are weaker.*

*In the GSEA report, a p value of zero (0.0) indicates an actual p value of less than 1/number-of-permutations. For example, if the analysis performed 100 permutations, a reported p value of 0.0 indicates an actual p value of less than 0.001. For a more accurate p value, increase the number of permutations performed by the analysis. Typically, you will want to perform 1000 permutations (phenotype or gene set). (If you attempt to perform significantly more than 1000 permutations, GSEA may run out of memory.)”*

**Table S8. Gene-based modified Sidak’s corrected *p*-values for the genes within the most strongly associated neurocognitive gene sets for the different SCZ GWASs and the NCNG sample.** The modified Sidak’s corrected *p*-values (i.e. minimum *p-*values from all the SNPs in the bin corrected for the number of SNPs in the bin) for the genes within the neurocognitive gene sets showing top enrichment in more than 2 samples for SCZ (i.e. colour-word interference - 25) are shown. For each gene, the Ensembl54 gene ID is shown (Ens54ID), followed by the chromosome position (Ens54Pos), gene symbol from the HUGO Gene Nomenclature Committee (HGNC), and gene name from the Database for Annotation, Visualization and Integrated Discovery v6.7. Please note that sets larger than 50 genes are not displayed here; they can be sent by email on request to the corresponding author.

| **Schizophrenia GWAS datasets** | | | | | **NCNG** | **German-Dutch** | **Danish** | **Norwegian TOP** | **PGC** |
| --- | --- | --- | --- | --- | --- | --- | --- | --- | --- |
| **Neurocognitive gene sets** | **Ens54ID** | **Ens54Pos** | **HGNC** | **DAVID** | ***p*-value** | ***p*-value** | ***p*-value** | ***p*-value** | ***p*-value** |
|  | ENSG00000134470 | chr10:6031259-6060156 | IL15RA | interleukin 15 receptor, alpha | 0.000037 | 0.058 | 0.89 | 0.19 | 0.84 |
|  | ENSG00000106025 | chr7:120214612-120285413 | TSPAN12 | tetraspanin 12 | 0.000054 | 0.090 | 0.86 | 0.87 | 0.052 |
| **Colour-word interference -25** | ENSG00000206951 | chr2:33023910-33024022 |  | hypothetical LOC100271832; RNA, Ro-associated Y5 pseudogene 10; RNA, Ro-associated Y1; RNA, Ro-associated Y4 pseudogene 7; RNA, Ro-associated Y4 pseudogene 19; RNA, Ro-associated Y3; hypothetical LOC100132111; RNA, Ro-associated Y4 | 0.000067 | 0.52 | 0.12 | 0.33 | 0.95 |
|  | ENSG00000148943 | chr11:27472700-27484879 | LIN7C | lin-7 homolog C (C, elegans) | 0.000070 | 0.20 | 0.94 | 0.53 | 0.53 |
|  | ENSG00000071243 | chr7:120378053-120402938 | ING3 | inhibitor of growth family, member 3 | 0.000083 | 0.099 | 0.96 | 0.94 | 0.069 |
|  | ENSG00000185641 | chr11:27559730-27560223 |  | ENSG00000185641 | 0.000093 | 0.14 | 0.072 | 0.88 | 0.30 |
|  | ENSG00000066855 | chr8:66719528-66785339 | MTFR1 | mitochondrial fission regulator 1 | 0.00010 | 0.87 | 0.86 | 0.89 | 0.050 |
|  | ENSG00000219943 | chr4:184539670-184539852 |  | ENSG00000219943 | 0.00014 | 0.0017 | 0.34 | 0.98 | 0.96 |
|  | ENSG00000176697 | chr11:27633016-27700181 | BDNF | brain-derived neurotrophic factor | 0.00016 | 0.19 | 0.67 | 0.94 | 0.42 |
| **Schizophrenia GWAS datasets (cont.)** | | | | | **NCNG** | **German-Dutch** | **Danish** | **Norwegian TOP** | **PGC** |
| **Neurocognitive gene sets** | **Ens54ID** | **Ens54Pos** | **HGNC** | **DAVID** | ***p*-value** | ***p*-value** | ***p*-value** | ***p*-value** | ***p*-value** |
| **Colour-word interference -25 (cont.)** | ENSG00000212289 | chr11:27499728-27499849 |  | RNA, 5S ribosomal 9; RNA, 5S ribosomal 13; RNA, 5S ribosomal 12; RNA, 5S ribosomal 11; RNA, 5S ribosomal 10; RNA, 5S ribosomal 17; RNA, 5S ribosomal 16; RNA, 5S ribosomal 15; RNA, 5S ribosomal 14; RNA, 5S ribosomal 1; RNA, 5S ribosomal 2; RNA, 5S ribosomal 3; RNA, 5S ribosomal 4; RNA, 5S ribosomal 5; RNA, 5S ribosomal 6; RNA, 5S ribosomal 7; RNA, 5S ribosomal 8 | 0.00011 | 0.14 | 0.98 | 0.71 | 0.32 |
|  | ENSG00000162644 | chr1:89342556-89343038 |  | ENSG00000162644 | 0.00031 | 0.97 | 0.96 | 0.98 | 0.98 |
|  | ENSG00000112308 | chr6:24813146-24828108 | C6orf62 | chromosome 6 open reading frame 62 | 0.00031 | 0.18 | 0.95 | 0.011 | 0.082 |
|  | ENSG00000209035 | chr3:102817361-102817461 |  | ENSG00000209035 | 0.00032 | 0.24 | 0.54 | 0.69 | 0.94 |
|  | ENSG00000173473 | chr3:47602391-47798410 | SMARCC1 | SWI/SNF related, matrix associated, actin dependent regulator of chromatin, subfamily c, member 1 | 0.00032 | 0.55 | 1.00 | 0.37 | 0.65 |
|  | ENSG00000015413 | chr16:88214501-88232365 | DPEP1 | dipeptidase 1 (renal) | 0.00033 | 0.75 | 0.55 | 0.87 | 0.0012 |
|  | ENSG00000111802 | chr6:24758185-24775094 | TTRAP | TRAF and TNF receptor associated protein | 0.00033 | 0.15 | 0.96 | 0.0026 | 0.015 |
|  | ENSG00000112304 | chr6:24775254-24809917 | THEM2 | acyl-CoA thioesterase 13 | 0.00035 | 0.529 | 0.99 | 0.020 | 0.025 |
|  | ENSG00000162645 | chr1:89345898-89364387 | GBP2 | guanylate binding protein 2, interferon-inducible | 0.00039 | 0.99 | 0.98 | 0.98 | 0.96 |
| **Schizophrenia GWAS datasets (cont.)** | | | | | **NCNG** | **German-Dutch** | **Danish** | **Norwegian TOP** | **PGC** |
| **Neurocognitive gene sets** | **Ens54ID** | **Ens54Pos** | **HGNC** | **DAVID** | ***p*-value** | ***p-*value** | ***p*-value** | ***p*-value** | ***p*-value** |
|  | ENSG00000183779 | chr8:37672467-37675554 | ZNF703 | zinc finger protein 703 | 0.00040 | 0.71 | 0.43 | n.t | 0.72 |
|  | ENSG00000081154 | chr3:102775732-102795969 | PCNP | PEST proteolytic signal containing nuclear protein | 0.00041 | 0.29 | 0.67 | 0.81 | 0.97 |
| **Colour-word intereference -25 (cont.)** | ENSG00000083457 | chr17:3564672-3651293 | ITGAE | integrin, alpha E (antigen CD103, human mucosal lymphocyte antigen 1; alpha polypeptide) | 0.00048 | 0.169 | 0.67 | 0.32 | 0.85 |
|  | ENSG00000204915 | chrX:45475521-45476189 |  | hypothetical LOC392452 | 0.00048 | 0.029 | 0.078 | 0.41 | n.t. |
|  | ENSG00000207725 | chrX:45491365-45491474 | MIRN222 | microRNA 222 | 0.00048 | 0.029 | 0.078 | 0.43 | n.t. |
|  | ENSG00000207870 | chrX:45490529-45490638 | MIRN221 | microRNA 221 | 0.00048 | 0.029 | 0.078 | 0.54 | n.t. |
|  | ENSG00000187821 | chr4:186177077-186178920 | HELT | HES/HEY-like transcription factor | 0.00054 | 0.73 | 0.0040 | 0.51 | 0.23 |

**Table S9. Gene-based modified Sidak’s corrected *p*-values for the genes within the most strongly associated neurocognitive gene sets for the different BPD GWASs and the NCNG sample.** The modified Sidak’s corrected *p*-values (i.e. minimum *p-*values from all the SNPs in the bin corrected for the number of SNPs in the bin) for the genes within the neurocognitive gene sets showing the most significant enrichment (i.e. visuospatial attention.1 - 25) and the neurocognitive gene sets showing enrichment in more than 2 samples for BPD (i.e. visuospatial attention.2 – 25, verbal abilities – 25, verbal abilities – 100 and delayed verbal memory - 1000) are shown. For each gene, the Ensembl54 gene ID is shown (Ens54ID), followed by the chromosome position (Ens54Pos), gene symbol from the HUGO Gene Nomenclature Committee (HGNC), and gene name from the Database for Annotation, Visualization and Integrated Discovery v6.7.

| **Bipolar Disorder GWAS datasets** | | | | | **NCNG** | **German** | **WTCCC** | **Norwegian TOP** | **PGC** |
| --- | --- | --- | --- | --- | --- | --- | --- | --- | --- |
|  |  |  |  |  |  |  |  |  |  |
| **Neurocognitive gene sets** | **Ens54ID** | **Ens54Pos** | **HGNC** | **DAVID** | ***p*-value** | ***p*-value** | ***p-*value** | ***p*-value** | ***p*-value** |
| **Visuospatial attention.1 -25** | ENSG00000076554 | chr8:81109662-81155565 | TPD52 | tumor protein D52 | 0.00013 | 0.26 | 0.22 | 0.078 | 0.92 |
|  | ENSG00000159263 | chr21:36993861-37044088 | SIM2 | single-minded homolog 2 (Drosophila) | 0.00017 | 0.65 | 0.84 | 0.20 | 0.97 |
|  | ENSG00000213368 | chr12:56664276-56664817 |  | ENSG00000213368 | 0.0002 | 0.35 | 0.95 | 0.97 | 0.90 |
|  | ENSG00000222210 | chr12:56658015-56658323 |  | Rn.t., 7SK small nuclear | 0.0002 | 0.35 | 0.94 | 0.96 | 0.90 |
|  | ENSG00000203987 | chr9:139882198-139906843 |  | hypothetical LOC100133077 | 0.00023 | 0.05 | 0.65 | 0.82 | 0.36 |
|  | ENSG00000219147 | chr6:25260753-25261725 |  | ENSG00000219147 | 0.00024 | 0.86 | 0.085 | 0.17 | 1.00 |
|  | ENSG00000220122 | chr6:25260754-25261726 |  | ENSG00000220122 | 0.00024 | 0.86 | 0.085 | 0.17 | 1.00 |
|  | ENSG00000221214 | chr10:112738674-112738761 | MIRN548E | ENSG00000221214 | 0.00025 | 0.0049 | 0.26 | 0.21 | 1.00 |
| **Bipolar Disorder GWAS datasets (cont.)** | | | | | **NCNG** | **German** | **WTCCC** | **Norwegian TOP** | **PGC** |
| **Neurocognitive gene sets** | **Ens54ID** | **Ens54Pos** | **HGNC** | **DAVID** | ***p*-value** | ***p*-value** | ***p-*value** | ***p*-value** | ***p*-value** |
|  | ENSG00000115009 | chr2:228386802-228390516 | CCL20 | chemokine (C-C motif) ligand 20 | 0.00026 | 0.82 | 0.034 | 0.20 | 0.95 |
|  | ENSG00000219126 | chr10:112686370-112686981 | RPL13AP6 | ribosomal protein L13a pseudogene 6 | 0.00026 | 0.0058 | 0.36 | 0.28 | 0.98 |
|  | ENSG00000166896 | chr12:56621627-56637318 | XRCC6BP1 | XRCC6 binding protein 1 | 0.00027 | 0.55 | 0.94 | 0.83 | 0.98 |
|  | ENSG00000179219 | chr16:83874065-83879184 | TMEM148 | transmembrane protein 148 | 0.0003 | 0.74 | 0.21 | 0.06 | 0.27 |
|  | ENSG00000171004 | chrX:131587719-131923093 | HS6ST2 | heparan sulfate 6-O-sulfotransferase 2 | 0.00033 | 0.93 | 0.32 | n.t. | n.t. |
|  | ENSG00000221185 | chr8:85370793-85370878 |  | ENSG00000221185 | 0.00033 | 0.82 | 0.80 | 0.38 | 0.37 |
| **Visuospatial attention.1 -25 (cont.)** | ENSG00000150593 | chr10:112621586-112649753 | PDCD4 | programmed cell death 4 (neoplastic transformation inhibitor) | 0.00038 | 0.0088 | 0.31 | 0.33 | 1.00 |
|  | ENSG00000214413 | chr10:112650120-112668934 | NCRn.t.00081 | non-protein coding Rn.t. 81 | 0.00038 | 0.0083 | 0.32 | 0.33 | 1.00 |
|  | ENSG00000189235 | chr6:25248191-25249628 |  | ENSG00000189235 | 0.00039 | 0.92 | 0.075 | 0.14 | 0.76 |
|  | ENSG00000219682 | chr6:25248210-25249607 |  | ENSG00000219682 | 0.00039 | 0.92 | 0.075 | 0.14 | 0.76 |
|  | ENSG00000170653 | chr12:52196111-52306405 | ATF7 | activating transcription factor 7 | 0.0004 | 0.46 | 0.071 | 0.33 | 0.97 |
|  | ENSG00000006128 | chr7:97199311-97207696 | TAC1 | tachykinin, precursor 1 | 0.00042 | 0.86 | 0.98 | 0.28 | 1.00 |
|  | ENSG00000134758 | chr18:27925835-27965521 | RNF138 | ring finger protein 138 | 0.00043 | 0.29 | 0.46 | 0.38 | 0.99 |
|  | ENSG00000214917 | chr18:27909848-27910233 |  | ENSG00000214917 | 0.00043 | 0.25 | 0.31 | 0.77 | 0.96 |
|  | ENSG00000108061 | chr10:112669357-112763412 | SHOC2 | soc-2 suppressor of clear homolog (C, elegans) | 0.00049 | 0.011 | 0.36 | 0.41 | 1.00 |
|  | ENSG00000207960 | chr7:157059789-157059875 | MIRN153-2 | microRn.t. 153-2 | 0.0005 | 0.90 | 0.38 | 0.97 | 0.10 |
| **Bipolar Disorder GWAS datasets (cont.)** | | | | | **NCNG** | **German** | **WTCCC** | **Norwegian TOP** | **PGC** |
| **Neurocognitive gene sets** | **Ens54ID** | **Ens54Pos** | **HGNC** | **DAVID** | ***p*-value** | ***p*-value** | ***p-*value** | ***p*-value** | ***p*-value** |
|  | ENSG00000222943 | chr6:73305004-73305091 |  | ENSG00000222943 | 0.00055 | 0.95 | 0.72 | 0.69 | 0.18 |
|  | ENSG00000202469 | chrX:9989262-9989363 |  | hypothetical LOC100271832; Rn.t., Ro-associated Y5 pseudogene 10; Rn.t., Ro-associated Y1; Rn.t., Ro-associated Y4 pseudogene 7; Rn.t., Ro-associated Y4 pseudogene 19; Rn.t., Ro-associated Y3; hypothetical LOC100132111; Rn.t., Ro-associated Y4 | 0.000026 | 0.037 | 0.77 | n.t. | n.t. |
|  | ENSG00000131409 | chr19:55711961-55763114 | LRRC4B | leucine rich repeat containing 4B | 0.000031 | 0.11 | 0.38 | 0.0046 | 0.27 |
| **Verbal abilities -50** | ENSG00000146243 | chr6:79633908-79665039 | IRAK1BP1 | interleukin-1 receptor-associated kin.t.se 1 binding protein 1 | 0.00004 | 0.0083 | 0.96 | 0.87 | 0.94 |
|  | ENSG00000146247 | chr6:79707008-79844708 | PHIP | pleckstrin homology domain interacting protein | 0.000047 | 0.056 | 0.89 | 0.91 | 0.95 |
|  | ENSG00000214684 | chr5:136221216-136221933 |  | ENSG00000214684 | 0.000086 | 0.91 | 0.96 | 0.95 | 1.00 |
|  | ENSG00000155158 | chr9:15161561-15297250 | TTC39B | tetratricopeptide repeat domain 39B | 0.000089 | 0.75 | 0.74 | 0.037 | 0.97 |
|  | ENSG00000155052 | chr2:124499334-125389382 | CNTn.t.P5 | contactin associated protein-like 5 | 0.00012 | 0.64 | 0.08478 | 0.027 | 0.048 |
|  | ENSG00000047644 | chrX:9943795-10072515 | WWC3 | WWC family member 3 | 0.00014 | 0.18 | 0.90 | n.t. | n.t. |
|  | ENSG00000053524 | chr3:184378525-184628549 | MCF2L2 | MCF,2 cell line derived transforming sequence-like 2 | 0.00015 | 0.89 | 0.33 | 0.68 | 0.12 |
| **Bipolar Disorder GWAS datasets (cont.)** | | | | | **NCNG** | **German** | **WTCCC** | **Norwegian TOP** | **PGC** |
| **Neurocognitive gene sets** | **Ens54ID** | **Ens54Pos** | **HGNC** | **DAVID** | ***p*-value** | ***p*-value** | ***p-*value** | ***p*-value** | ***p*-value** |
|  | ENSG00000187772 | chr6:105511616-105637899 | LIN28B | lin-28 homolog B (C, elegans) | 0.00015 | 0.44 | 0.67 | 0.85 | 0.87 |
|  | ENSG00000217512 | chr6:79834128-79834574 |  | ENSG00000217512 | 0.00016 | 0.021 | 0.85 | 0.51 | 0.97 |
|  | ENSG00000125817 | chr20:3712498-3715337 | CENPB | centromere protein B, 80kDa | 0.00018 | 0.80 | 0.00013 | 0.83 | 0.096 |
|  | ENSG00000101222 | chr20:3706152-3710102 | SPEF1 | sperm flagellar 1 | 0.00022 | 0.67 | 0.00013 | 0.83 | 0.11 |
|  | ENSG00000163623 | chr4:85633460-85638411 | NKX6-1 | NK6 homeobox 1 | 0.00024 | 0.35 | 0.39 | 0.92 | 0.76 |
|  | ENSG00000159842 | chr17:853510-1029881 | ABR | active BCR-related gene | 0.00024 | 0.37 | 0.47 | 0.78 | 0.026 |
|  | ENSG00000136848 | chr9:123368983-123587630 | DAB2IP | DAB2 interacting protein | 0.00025 | 0.25 | 0.62 | 0.45 | 0.51 |
| **Verbal abilities -50 (cont.)** | ENSG00000074211 | chr4:6373214-6525227 | PPP2R2C | protein phosphatase 2 (formerly 2A), regulatory subunit B, gamma isoform | 0.00025 | 0.23 | 0.39 | 0.95 | 0.68 |
|  | ENSG00000200201 | chr11:3642969-3643065 |  | hypothetical LOC100271832; Rn.t., Ro-associated Y5 pseudogene 10; Rn.t., Ro-associated Y1; Rn.t., Ro-associated Y4 pseudogene 7; Rn.t., Ro-associated Y4 pseudogene 19; Rn.t., Ro-associated Y3; hypothetical LOC100132111; Rn.t., Ro-associated Y4 | 0.00029 | 0.032 | 0.41 | 0.15 | 0.72 |
|  | ENSG00000209396 | chr11:3669853-3669949 |  | ENSG00000209396 | 0.00029 | 0.0089 | 0.34 | 0.51 | 0.96 |
| **Bipolar Disorder GWAS datasets (cont.)** | | | | | **NCNG** | **German** | **WTCCC** | **Norwegian TOP** | **PGC** |
| **Neurocognitive gene sets** | **Ens54ID** | **Ens54Pos** | **HGNC** | **DAVID** | ***p*-value** | ***p*-value** | ***p-*value** | ***p*-value** | ***p*-value** |
|  | ENSG00000129749 | chr11:3643394-3649190 | CHRn.t.10 | cholinergic receptor, nicotinic, alpha 10 | 0.00039 | 0.042 | 0.41 | 0.16 | 0.79 |
|  | ENSG00000101220 | chr20:3682155-3704386 | C20orf27 | chromosome 20 open reading frame 27 | 0.0004 | 0.59 | 0.00018 | 0.27 | 0.19 |
|  | ENSG00000201279 | chr11:3641621-3641717 |  | hypothetical LOC100271832; Rn.t., Ro-associated Y5 pseudogene 10; Rn.t., Ro-associated Y1; Rn.t., Ro-associated Y4 pseudogene 7; Rn.t., Ro-associated Y4 pseudogene 19; Rn.t., Ro-associated Y3; hypothetical LOC100132111; Rn.t., Ro-associated Y4 | 0.00029 | 0.032 | 0.41 | 0.15 | 0.72 |
| **Verbal abilities -50 (cont.)** | ENSG00000101224 | chr20:3724401-3734757 | CDC25B | cell division cycle 25 homolog B (S, pombe) | 0.00044 | 0.54 | 0.00024 | 0.13 | 0.26 |
|  | ENSG00000210188 | chr14:72577080-72577374 |  | ENSG00000210188 | 0.00045 | 0.038 | 0.11 | 0.36 | 1.00 |
|  | ENSG00000222645 | chr14:72577083-72577375 |  | Rn.t., 7SL, cytoplasmic 2; Rn.t., 7SL, cytoplasmic 1 | 0.00045 | 0.038 | 0.11 | 0.36 | 1.00 |
|  | ENSG00000129744 | chr11:3622937-3642222 | ART1 | ADP-ribosyltransferase 1 | 0.00046 | 0.049 | 0.45 | 0.19 | 0.87 |
|  | ENSG00000003147 | chr7:8119339-8268767 | ICA1 | islet cell autoantigen 1, 69kDa | 0.00046 | 0.37 | 0.55 | 0.12 | 0.19 |
|  | ENSG00000170369 | chr20:23752406-23755368 | CST2 | cystatin SA | 0.00047 | 0.81 | 0.062 | 0.95 | 0.87 |
|  | ENSG00000197830 | chr5:137910194-137910566 |  | ENSG00000197830 | 0.00051 | 0.95 | 0.10 | 0.99 | 0.10 |
| **Bipolar Disorder GWAS datasets (cont.)** | | | | | **NCNG** | **German** | **WTCCC** | **Norwegian TOP** | **PGC** |
| **Neurocognitive gene sets** | **Ens54ID** | **Ens54Pos** | **HGNC** | **DAVID** | ***p*-value** | ***p*-value** | ***p-*value** | ***p*-value** | ***p*-value** |
|  | ENSG00000219740 | chr1:170984323-170985265 |  | ENSG00000219740 | 0.00056 | 0.37 | 0.85 | 0.42 | 0.92 |
|  | ENSG00000218696 | chr13:48961107-48961501 |  | ENSG00000218696 | 0.00056 | 0.78 | 0.24 | 0.66 | 1.00 |
|  | ENSG00000219158 | chr19:59424723-59425900 |  | ENSG00000219158 | 0.00058 | 0.18 | 0.57 | 0.25 | 0.95 |
| **Verbal abilities -50 (cont.)** | ENSG00000202526 | chr1:226839466-226839584 | RN5S17 | Rn.t., 5S ribosomal 9; Rn.t., 5S ribosomal 13; Rn.t., 5S ribosomal 12; Rn.t., 5S ribosomal 11; Rn.t., 5S ribosomal 10; Rn.t., 5S ribosomal 17; Rn.t., 5S ribosomal 16; Rn.t., 5S ribosomal 15; Rn.t., 5S ribosomal 14; Rn.t., 5S ribosomal 1; Rn.t., 5S ribosomal 2; Rn.t., 5S ribosomal 3; Rn.t., 5S ribosomal 4; Rn.t., 5S ribosomal 5; Rn.t., 5S ribosomal 6; Rn.t., 5S ribosomal 7; Rn.t., 5S ribosomal 8 | 0.00059 | 0.081 | 0.86 | 0.96 | 0.91 |
|  | ENSG00000212502 | chr12:107696049-107696176 |  | small nucleolar Rn.t., H/ACA box 40 | 0.00059 | 0.061 | 0.81 | 0.50 | 0.99 |
|  | ENSG00000113013 | chr5:137918923-137939014 | HSPA9 | heat shock 70kDa protein 9 (mortalin) | 0.00061 | 0.94 | 0.10 | 0.99 | 0.11 |
|  | ENSG00000206989 | chr5:137924631-137924698 | SNORD63 | small nucleolar Rn.t., C/D box 63 | 0.00061 | 0.94 | 0.10 | 0.99 | 0.11 |
|  | ENSG00000222937 | chr5:137922558-137922627 |  | small nucleolar Rn.t., C/D box 63 | 0.00061 | 0.94 | 0.10 | 0.99 | 0.11 |
| **Bipolar Disorder GWAS datasets (cont.)** | | | | | **NCNG** | **German** | **WTCCC** | **Norwegian TOP** | **PGC** |
| **Neurocognitive gene sets** | **Ens54ID** | **Ens54Pos** | **HGNC** | **DAVID** | ***p*-value** | ***p*-value** | ***p-*value** | ***p*-value** | ***p*-value** |
|  | ENSG00000200312 | chr14:89248608-89248922 |  | Rn.t., 7SK small nuclear | 0.00061 | 0.0097 | 0.86 | 0.98 | 0.61 |
|  | ENSG00000220413 | chr1:240061860-240062712 |  | ENSG00000220413 | 0.00067 | 0.23 | 0.57 | 0.51 | 0.99 |
|  | ENSG00000186152 | chr19:59411959-59419190 | LILRB3 | leukocyte immunoglobulin-like receptor, subfamily B (with TM and ITIM domains), member 3 | 0.00068 | 0.72 | 0.65 | 0.41 | 0.95 |
|  | ENSG00000136169 | chr13:48916511-48964298 | SETDB2 | SET domain, bifurcated 2 | 0.0007 | 0.85 | 0.26 | 0.72 | 1.00 |
|  | ENSG00000118193 | chr1:198787930-198856485 | KIF14 | kinesin family member 14 | 0.00071 | 0.98 | 0.56 | 0.23 | 0.01 |
| **Verbal abilities -50 (cont.)** | ENSG00000215462 | chr13:48905496-48918555 |  | ENSG00000215462 | 0.00077 | 0.87 | 0.19 | 0.66 | 1.00 |
|  | ENSG00000216137 | chr1:206367194-206367263 |  | ENSG00000216137 | 0.00077 | 0.57 | 0.73 | 0.051 | 0.60 |
|  | ENSG00000198963 | chr9:76302072-76491937 | RORB | RAR-related orphan receptor B | 0.00077 | 0.83 | 0.32 | 0.69 | 0.89 |
|  | ENSG00000182836 | chr5:41342805-41546487 | PLCXD3 | phosphatidylinositol-specific phospholipase C, X domain containing 3 | 0.00078 | 0.23 | 0.71 | 0.032 | 0.22 |
|  | ENSG00000210543 | chr17:37139905-37140183 |  | ENSG00000210543 | 0.00079 | 0.60 | 0.27 | 0.35 | 0.96 |
|  | ENSG00000110713 | chr11:3652817-3775468 | NUP98 | nucleoporin 98kDa | 0.00081 | 0.014 | 0.73 | 0.60 | 0.93 |
|  | ENSG00000219592 | chr21:27786327-27786451 |  | ENSG00000219592 | 0.00084 | 0.81 | 0.35 | 0.22 | 0.70 |
|  | | | | |  |  |  |  |  |
| **Bipolar Disorder GWAS datasets (cont.)** | | | | | **NCNG** | **German** | **WTCCC** | **Norwegian TOP** | **PGC** |
| **Neurocognitive gene sets** | **Ens54ID** | **Ens54Pos** | **HGNC** | **DAVID** | ***p*-value** | ***p*-value** | ***p*-value** | ***p*-value** | ***p*-value** |
| **Verbal abilities -50 (cont.)** | ENSG00000201920 | chr17:37127932-37128038 |  | Rn.t., 5S ribosomal 9; Rn.t., 5S ribosomal 13; Rn.t., 5S ribosomal 12; Rn.t., 5S ribosomal 11; Rn.t., 5S ribosomal 10; Rn.t., 5S ribosomal 17; Rn.t., 5S ribosomal 16; Rn.t., 5S ribosomal 15; Rn.t., 5S ribosomal 14; Rn.t., 5S ribosomal 1; Rn.t., 5S ribosomal 2; Rn.t., 5S ribosomal 3; Rn.t., 5S ribosomal 4; Rn.t., 5S ribosomal 5; Rn.t., 5S ribosomal 6; Rn.t., 5S ribosomal 7; Rn.t., 5S ribosomal 8 | 0.00079 | 0.60 | 0.27 | 0.35 | 0.97 |
|  | ENSG00000140853 | chr16:55580911-55674937 | NLRC5 | NLR family, CARD domain containing 5 | 0.000035 | 0.17 | 0.098 | 0.62 | 0.057 |
| **Delayed verbal memory -25** | ENSG00000208022 | chr12:79853646-79853743 | MIRN618 | microRn.t. 618 | 0.000043 | 0.007 | 0.021 | 0.81 | 0.99 |
|  | ENSG00000203357 | chr12:79854446-79855824 |  | ENSG00000203357 | 0.000055 | 0.0089 | 0.032 | 0.64 | 0.95 |
|  | | | | |  |  |  |  |  |
| **Bipolar Disorder GWAS datasets (cont.)** | | | | | **NCNG** | **German** | **WTCCC** | **Norwegian TOP** | **PGC** |
| **Neurocognitive gene sets** | **Ens54ID** | **Ens54Pos** | **HGNC** | **DAVID** | ***p-*value** | ***p*-value** | ***p*-value** | ***p*-value** | ***p-*value** |
|  | ENSG00000176971 | chr11:26972204-26975206 | FIBIN | fin bud initiation factor homolog (zebrafish) | 0.00014 | 0.39331 | 0.01 | 0.49 | 1.00 |
|  | ENSG00000111052 | chr12:79715306-79855825 | LIN7A | lin-7 homolog A (C, elegans) | 0.00017 | 0.026 | 0.10 | 0.98 | 0.94 |
|  | ENSG00000186210 | chr4:78099038-78099340 |  | ENSG00000186210 | 0.00021 | 0.89 | 0.99 | 0.45 | 0.98 |
|  | ENSG00000115350 | chr2:75039283-75050366 | POLE4 | polymerase (Dn.t.-directed), epsilon 4 (p12 subunit) | 0.0003 | 0.97 | 0.14 | 0.37 | 0.75 |
|  | ENSG00000204792 | chr2:75008874-75013659 |  | ENSG00000204792 | 0.00033 | 0.96 | 0.15 | 0.43 | 0.81 |
|  | ENSG00000108639 | chr17:73676266-73680604 | SYNGR2 | syn.t.ptogyrin 2 | 0.00037 | 0.30 | 0.19 | 0.93 | 0.25 |
|  | ENSG00000210110 | chr20:30059831-30059901 |  | ENSG00000210110 | 0.00039 | 0.36 | 0.51 | 0.57 | 0.98 |
| **Delayed verbal memory -25** | ENSG00000167900 | chr17:73681755-73694909 | TK1 | thymidine kin.t.se 1, soluble | 0.00043 | 0.35 | 0.11 | 0.83 | 0.28 |
|  | ENSG00000183077 | chr17:73694993-73715377 | AFMID | arylformamidase | 0.00043 | 0.50 | 0.086 | 0.18 | 0.30 |
|  | ENSG00000187997 | chr17:73654029-73673829 | C17orf99 | chromosome 17 open reading frame 99 | 0.00052 | 0.39 | 0.07 | 0.58 | 0.41 |
|  | ENSG00000197079 | chr17:36886467-36890918 | KRT35 | keratin 35 | 0.00053 | 0.44 | 0.85 | 1.00 | 0.14 |
|  | ENSG00000138758 | chr4:78089919-78178791 | 40787 | septin 11 | 0.00053 | 1.00 | 0.99 | 0.46 | 0.99 |
|  | ENSG00000198440 | chr19:61607530-61628212 | ZNF583 | zinc finger protein 583 | 0.00055 | 0.82 | 0.036 | 0.79 | 0.60 |
|  | ENSG00000178343 | chr4:42094613-42099261 | SHISA3 | shisa homolog 3 (Xenopus laevis) | 0.00059 | 0.59 | 0.14 | 0.67 | 0.06 |
|  | ENSG00000220315 | chr1:20891814-20893093 |  | ENSG00000220315 | 0.00065 | 0.32 | 0.33 | 0.45 | 0.83 |
|  | ENSG00000126337 | chr17:36895916-36902324 | KRT36 | keratin 36 | 0.00065 | 0.52 | 0.92 | 1.00 | 0.16 |
| **Bipolar Disorder GWAS datasets (cont.)** | | | | | **NCNG** | **German** | **WTCCC** | **Norwegian TOP** | **PGC** |
| **Neurocognitive gene sets** | **Ens54ID** | **Ens54Pos** | **HGNC** | **DAVID** | ***p*-value** | ***p-*value** | ***p*-value** | ***p*-value** | ***p-*value** |
|  | ENSG00000170873 | chr8:125632212-125809911 | MTSS1 | metastasis suppressor 1 | 0.00069 | 0.66 | 0.56 | 0.41 | 0.60 |
|  | ENSG00000223125 | chr17:36877734-36877924 |  | Rn.t., U2 small nuclear 1; Rn.t., U2 small nuclear 2 | 0.00069 | 0.54 | 0.94 | 1.00 | 0.18 |
| **Delayed verbal memory -25 (cont.)** | ENSG00000211366 | chr9:90179004-90179092 |  | ENSG00000211366 | 0.00069 | 0.68 | 0.86 | 0.75 | 1.00 |
|  | ENSG00000208875 | chr1:146183362-146183433 |  | ENSG00000208875 | 0.00075 | 0.61 | 0.83 | 0.95 | 0.71 |
|  | ENSG00000219731 | chr1:146185064-146185580 |  | hypothetical LOC100134101 | 0.00075 | 0.61 | 0.83 | 0.95 | 0.71 |
|  | ENSG00000140030 | chr14:87541249-87548164 | GPR65 | G protein-coupled receptor 65 | 0.00075 | 0.93 | 0.19 | 0.29 | 1.00 |

**Table S10.** **Overlap between the most enriched cognitive genes sets in the PGC SCZ and BPD**

Here we report the average FDR *q*-values of the 10 most enriched cognitive gene sets in the PGC sample for SCZ and BPD. Considering the enrichment rank of the gene sets, colour-word interference, verbal abilities and visuospatial attention represent the most enriched sets that overlap between the disorders.

| **TOP 10 SCZ gene sets** | **FDR *q*-val** | **TOP 10 BPD gene sets** | **FDR *q*-val** |
| --- | --- | --- | --- |
| Learning -250 | 0.13 | Verbal abilities -50 | 0.42 |
| Colour-word interference -25 | 0.34 | Delayed verbal memory -100 | 0.54 |
| Visuospatial attention.1 -25 | 0.35 | Colour-word interference -50 | 0.58 |
| Learning -500 | 0.48 | Colour-word interference -25 | 0.62 |
| Verbal abilities -750 | 0.53 | Verbal abilities -250 | 0.62 |
| Estimated IQ -1000 | 0.53 | Matrix Reasoning -25 | 0.63 |
| Learning -1750 | 0.53 | Verbal abilities -25 | 0.71 |
| Visuospatial attention.1 -1000 | 0.54 | Verbal abilities -100 | 0.73 |
| Estimated IQ -1500 | 0.54 | Visuospatial attention.2 -25 | 0.73 |
| Visuospatial attention.3 -500 | 0.54 | Visuospatial attention.1 -25 | 0.78 |

**References**

1. Espeseth T, Christoforou A, Lundervold AJ, Steen VM, Le Hellard S, et al. (2012) Imaging and cognitive genetics: the Norwegian Cognitive NeuroGenetics sample. Twin Res Hum Genet 15: 442-452.

2. Wechsler D (1999) Weschler Abbreviated Scale of Intelligence: San Antonio TX: The Psychological Corporation.

3. Delis DC, Kramer JH, Kaplan E, Ober BA (2000) California Verbal Learning Test: San Antonio TX: The Psychological Corporation.

4. Delis DC, Kaplan E, Kramer JH (2001) D-KEFS: Examiners Manual: San Antonio TX: The Psychological Corporation.

5. Parasuraman R, Greenwood PM, Haxby JV, Grady CL (1992) Visuospatial attention in dementia of the Alzheimer type. Brain 115: 711-733.

6. Spreen O, Strauss E (1998) A compendium of neuropsychological tests: Administration, norms, and commentary: New York: Oxford University Press.

7. Folstein MF, Folstein SE, McHugh PR (1975) “Mini-mental state”: A practical method for grading the cognitive state of patients for the clinician. J Psychiatr Res 12: 189-198.

8. Beck AT, Ward CH, Mendelson M, Mock J, Erbaugh J (1961) An inventory for measuring depression. Arch Gen Psychiatry 4: 561-571.

9. Guo X, Liu Z, Wang X, Zhang H (2013) Genetic association test for multiple traits at gene level. Genet Epidemiol 37: 122-129.

10. Ersland KM, Christoforou A, Stansberg C, Espeseth T, Mattheisen M, et al. (2012) Gene-based analysis of regionally enriched cortical genes in GWAS data sets of cognitive traits and psychiatric disorders. PLoS One 7: e31687.

11. Warrington JA, Nair A, Mahadevappa M, Tsyganskaya M (2000) Comparison of human adult and fetal expression and identification of 535 housekeeping/maintenance genes. Physiol Genomics 2: 143–147.

12. Wellcome Trust Case Control Consortium (2007) Genome-wide association study of 14,000 cases of seven common diseases and 3,000 shared controls. Nature 447: 661-678.

13. Subramanian A, Tamayo P, Mootha VK, Mukherjee S, Ebert BL, et al. (2005) Gene set enrichment analysis: A knowledge-based approach for interpreting genome-wide expression profiles. Proc Natl Acad Sci U S A 102: 15545-15550.
